# The shaping role of self-organization: linking vegetation patterning, plant traits and ecosystem functioning

**DOI:** 10.1098/rspb.2018.2859

**Published:** 2019-04-10

**Authors:** Li-Xia Zhao, Chi Xu, Zhen-Ming Ge, Johan van de Koppel, Quan-Xing Liu

**Affiliations:** 1 State Key Laboratory of Estuarine and Coastal Research, School of Ecological and Environmental Sciences, East China Normal University, Shanghai 200241, People's Republic of China; 2 School of Life Sciences, Nanjing University, Nanjing 210023, China; 3 Department of Estuarine and Delta Systems, Royal Netherlands Institute for Sea Research and Utrecht University, PO Box 140, 4400 AC Yerseke, The Netherlands; 4 Shanghai Key Lab for Urban Ecological Processes and Eco-Restoration & Tiantong National Station for Forest Ecosystem Research, School of Ecological and Environmental Sciences, East China Normal University, Shanghai 200241, People's Republic of China; 5 Center for Global Change and Ecological Forecasting, School of Ecological and Environmental Science, East China Normal University, 200241 Shanghai, People's Republic of China

**Keywords:** spatial self-organization, scale-dependent feedback, irregular patterns, *Scirpus mariqueter*, ecosystems functioning, salt marsh

## Abstract

Self-organized spatial patterns are increasingly recognized for their contribution to ecosystem functioning, in terms of enhanced productivity, ecosystem stability, and species diversity in terrestrial as well as marine ecosystems. Most studies on the impact of spatial self-organization have focused on systems that exhibit regular patterns. However, there is an abundance of patterns in many ecosystems which are not strictly regular. Understanding of how these patterns are formed and how they affect ecosystem function is crucial for the broad acceptance of self-organization as a keystone process in ecological theory. Here, using transplantation experiments in salt marsh ecosystems dominated by *Scirpus mariqueter*, we demonstrate that scale-dependent feedback is driving irregular spatial pattern formation of vegetation. Field observations and experiments have revealed that this self-organization process affects a range of plant traits, including shoot-to-root ratio, rhizome orientation, rhizome node number, and rhizome length, and enhances vegetation productivity. Moreover, patchiness in self-organized salt marsh vegetation can support a better microhabitat for macrobenthos, promoting their total abundance and spatial heterogeneity of species richness. Our results extend existing concepts of self-organization and its effects on productivity and biodiversity to the spatial irregular patterns that are observed in many systems. Our work also helps to link between the so-far largely unconnected fields of self-organization theory and trait-based, functional ecology.

## 1. Introduction

In nature, organisms extended in space often form some kind of patterns rather than random distributions. Particular interest has been given to the patterns that exhibit conspicuous spatial regularities, ranging from fractal-like microbial colonies in Petri dishes [1,2] to evenly spaced termite mounds in dryland landscapes [3–6]. Among the most studied are the so-called ‘Turing patterns’ (a class of patterns with distinct periodicity in space, resembling regular spots, stripes, or labyrinths) that have been widely observed in many ecosystems such as dryland vegetation [7–9], peat bogs [10], and coastal mussel beds [11–13]. While these ecosystems are distinct in many respects, the formation of their distinctive Turing-like patterns could be driven by universal mechanisms, including scale-dependent feedback (SDF, referred to as coupled short-range positive feedbacks and long-range negative feedbacks; see [14,15]) and behavioural-driven phase separation [16,17]. These mechanisms are essentially at the core of self-organization processes [14,15,18–20], the important role of which has been increasingly demonstrated at all levels of organisms, from molecules to ecosystems [12,19,21–23].

Regular vegetation patterns (e.g. the tiger-bush in African drylands) have inspired the seminal work on ecosystem-level self-organization [7,24]. Intensive studies have converged to suggest that SDF is so-far the most common (but not the only [25,26]) mechanism underpinning regular patterns of patchy vegetation. While such patterns provide a clearly identifiable spatial sign for self-organized vegetation, an intriguing question is whether self-organization processes (SDF in particular) would necessarily give rise to Turing-like regular vegetation patterns. Little theoretical work has suggested that irregular spatial patterns can also arise under the conditions that SDF is at play (e.g. specified by short-range facilitation and long-range competition between plants in dryland ecosystems), as seen in both cellular automaton [27–29] and individual-based models [30]. However, field evidence is lacking in most ecosystems (but see [31,32]). A major barrier is that it is notoriously difficult to identify and quantify feedback interactions as well as their operational scales in real-world ecosystems. Another possible reason is that irregular vegetation patterns often receive less attention than their regular counterparts, though irregular patterns have a much wider distribution range, and contain equally important information for elucidating relevant ecological processes underlying pattern formation [29,32]. So far, the link between irregular vegetation patterning and self-organization remains elusive.

In parallel with the line of work on the mechanisms of self-organization, an emerging research interest involves if and how self-organization can influence ecosystem functioning. It has been speculated that self-organized ecosystems can be more ‘robust’, in terms of enhanced productivity, biodiversity, stability, and resilience [33,34]. This speculation has gained support from theoretical studies [11,13,35–37], but has rarely been verified in the field. Recent work has started to fill this gap, suggesting that self-organized ecosystems with regular patterns are indeed associated with enhanced productivity and stability [3,38,39]. If promotion of ecosystem functioning is a necessary outcome of self-organization, an important corollary is that self-organization, if present, may also enhance the functioning of irregularly patterned ecosystems which are widespread in nature.

In this study, we combined remote sensing, field observations, and *in situ* experiments to demonstrate that self-organization may play an important role in the irregular vegetation patterning of an intertidal salt marsh of China. We reveal that the dominant plant species, *Scirpus mariqueter*, as an ecosystem engineer is self-organized through SDF to form a patchy vegetation structure. We further show that this self-organization process is associated with modifications of a range of plant functional traits [40] as well as enhanced vegetation productivity. We also find that the self-organized salt marsh vegetation can create a better microhabitat for macrobenthos, promoting their total abundance and spatial heterogeneity of species richness. Our work highlights the possibility that self-organization plays a more widespread role in ecosystem patterning and functioning than previously thought. It also helps to build a holistic picture on how these fundamental attributes of ecosystems at different levels (including plant traits, vegetation patterning, and ecosystem functioning) are inter-linked via self-organization.

## 2. Material and methods

### (a) Field sites and hypotheses

Our study was conducted in the Chongming Dongtan Nature Reserve on the eastern Chongming Island, the largest alluvial island (sized of *ca* 1267 km^2^) in the Yangtze estuary, China. Our study site (31°27′31.86720″ N, 121°55′48.95760″ E) is located on the intertidal mudflat influenced by semidiurnal tides. *Phragmites australis* and *S. mariqueter* are the dominant species in the salt marsh ecosystems. Our experiments and field investigations were implemented in the tidal front zone, where *S. mariqueter* is the dominant pioneer plant species that colonizes this highly stressful environment.

**Figure 1.**
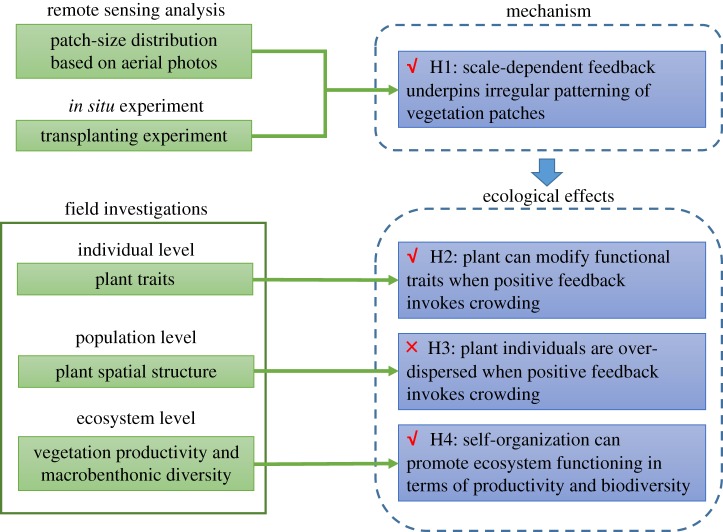
The flowchart of study approach (left column) and hypothesis testing on the mechanism and ecological effects of self-organization (right column). Symbols √ and×indicate working hypotheses that are accepted and rejected by the results, respectively.

While the spatial arrangement of the vegetation patches (dominated by *S. mariqueter*) in the study area seemingly deviate from regular patterns, we strictly tested if the irregularity holds with a patch-size distribution analysis. Our first working hypothesis is that SDF exists in the irregular (if supported by the above-mentioned test) vegetation patterning. We tested this hypothesis through *in situ* transplant experiments that can provide direct evidence if existing vegetation patches can have a positive effect at a smaller scale (short-range positive feedback) and a negative effect at a larger scale (long-range negative feedback) on transplanted individuals (in terms of survival and growth). If the transplant experiments can indeed support the existence of SDF, we then ask if SDF can modify functional traits and spatial structure of individual plants. Since positive feedbacks would by all means lead to increasing local crowdedness, therefore better ability of resistance against wave impacts and meanwhile rising tension of competition between plant individuals, we hypothesize that plants would respond through modifications of their traits (e.g. allocating less below-ground biomass) and micro-scale structure (e.g. over-dispersion to avoid much overlap between individuals). We tested this hypothesis through field survey on plant traits and spatial analysis on microstructure. Our last hypothesis is that self-organization (if present as indicated by the existence of SDF) can affect ecosystem functioning. We used the biodiversity and primary productivity of the macrobenthos as a measure of the effect of SDF on ecosystem functioning and collected data in the field to test this hypothesis. Although biodiversity is usually considered as a driver, here we adopt biodiversity as a measure of ecosystem functioning to align with previous self-organization studies [41]. See figure 1 for the framework of hypothesis testing and the roadmap of data collection and analyses in this study.

### (b) Remote sensing analysis

We used very high-resolution (1.3 cm) aerial photos taken by an unmanned aerial vehicle (UAV) to map the spatial distribution of *S. mariqueter* patches. We analysed the patch-size distribution (i.e. frequency distribution of patch size) to check whether the vegetation follows a normal distribution as expected for a regular pattern. This analysis was conducted based on six randomly selected rectangle areas typical of the studied vegetation (five areas sized 100 × 100 m^2^ and one sized 200 × 200 m^2^; these images cover a total area of 2 km^2^), see electronic supplementary material, figure S1. Before analysis, we excluded incomplete patches (intersecting with the boundary) to avoid the edge effect. As the first step, we checked the resulting histograms and used kernel density estimation to illustrate the probability density distribution of patch size. We then fitted ordinary least-square linear models using logarithmic bins to infer if the emerging fat-tailed distributions (see Results and discussion) follow a power law (scale-free pattern) [11,29,42,43]. While this method has been criticized for biased exponent estimation [44], it is sufficiently robust for a qualitative identification of power-law-like, non-regular distribution as we found in this study.

### (c) Transplanting experiment

SDF has been commonly observed in the formation of patchy vegetation structure in stressful environments such as coastal salt marshes [31,45]. In general, it is difficult to fully solidify the existence of SDF in the field experiments, as patch development is a long-term process. To date, transplanting experiments have provided the most convincing evidence *in situ* of the existence of SDF [31]. For example, by comparing the growth of transplanted plant units between inside (short-range interactions) and outside (long-range interactions) established vegetation patches, van Wesenbeeck *et al*. 2008 [31] demonstrated enhanced performance within patches while suppressed growth in the sediments next to the patches for the pioneer plant species *Spartina angilica* in a Dutch intertidal salt marsh ecosystem, indicating short-range positive feedback and long-range negative feedback. In this study, we conducted 2-year (in 2017 and 2018) transplant experiments that are similar to that of van Wesenbeeck *et al*. 2008 [31], to test if SDF shapes *S. mariqueter* patches in our study site. For each patch pair, we transplanted small *S. mariqueter* at the inter-patch gap and around the centres of both patches along a straight line (representing three treatments). For all treatments, we transplanted *S. mariqueter* (obtained from neighbouring homogeneous *S. mariqueter* vegetated areas) within a consistent sediment sample volume of 50 × 50 × 20 cm^3^ in May 2017 and June 2018, respectively. All transplanting plots were flagged with PVC tubes at their corners. In the 2018 experiment, we transplanted *S. mariqueter* at the centre (0 m), close to (1 m), and faraway from the patch (5 m), and added a control treatment to account for the potential influence of competition effect from neighbouring plants. The control treatment is meant to account for intraspecific competition of *S. mariqueter*. In the studied intertidal salt marsh where nutrient supply is ample [46,47], competition for light may play an important role in shaping vegetation structure [45]. In response to light competition, plants may promote vertical growth and shade tolerance to enhance their competitiveness, or alleviate the tension of competition through lateral growth [48]. To exclude the potential influence of light competition on the test of SDF, we implemented the control treatment next to the within-patch (0 m) treatment, close to the patch centre. We removed the above-ground part of the neighbouring plants within a distance of 20 cm to the borders of the transplanted plots every two weeks (see the electronic supplementary material, figure S3c). Every week we measured stem density within the transplanted plots at the three distances (i.e. 0 m, 1 m, and 5 m) as well as within the control treatment plots. After the 2018 experiment, four out of six replicate plots remained intact (*n* = 4 for statistical analysis).

### (d) Field investigations

Spatial heterogeneity in the studied landscape is mostly attributed to wave stress and vegetation cover. To account for the physical stress of tides, we distinguished between the areas that are subject to different intensity of wave force. ‘Exposed areas’ are referred to as low tidal areas subject to strong wave impact, whereas ‘unexposed areas’ are referred to as high tidal areas with rather weak wave impact. There was a difference of 15–30 min per tidal cycle between the exposed and unexposed areas. In addition, we distinguished between the areas that harbour high and low biomass densities of *S. mariqueter*. Colonization of plants in high-density areas has often preceded that in their low-density counterparts. It has been documented that the intensity of short-range positive feedbacks (if present) could be a function of the biomass of established plants in intertidal ecosystems [49,50]. We therefore compared locations with different densities of *S. mariqueter* as a proxy for the strength of self-organization (a higher density corresponds with stronger self-organization). The high and low densities are about 4000 and 400 shoots per square metre, respectively. Considering these two factors, our field investigations were set up with four treatments, i.e. exposed areas with high plant densities, exposed areas with low plant densities, unexposed areas with high plant densities, unexposed areas with low plant densities. These treatments were distributed in a relatively homogeneous landscape spanning *ca* 2 km along the coastal line.

In order to study the effects of self-organized patterning on plant traits, we conducted detailed investigations on the spatial structure of *S. mariqueter* ramets as well as their functional traits in the field in September 2016. Within a randomly chosen 0.5 × 0.5 m^2^ vegetated quadrat of the four treatments mentioned above, we recorded the relative positions of all above-ground ramets to analyse their spatial patterns at small scale by means of the spatial pair-correlation function; meanwhile, we carefully dug out all below-ground rhizomes and retrieved the above-ground ramets that they connected. In this way, we investigated rhizome length of connected individuals (distance), number of branches of ramets (node number), and relative angle of all individuals connected to the same individuals (orientations). These functional traits are essential to the survival (and thereby fitness) of *S. mariqueter* [40], in the sense that they determine density and spatial arrangement of ramets, and therefore have important consequences on, for example, resource use strategy, between-ramet interactions, and resistance to stress (for example, high density may help to ameliorate wave impacts, but could reduce light and nutrient availability per ramet due to local crowding). It has been shown that self-organization could give rise to multi-scale patterning in mussel bed systems [13]. Here, we also tested if individual plants within the patches could have a non-random spatial structure resulting from self-organization of vegetation patches. If self-organization is at play, the short-range positive feedback would increase local density, and therefore one may expect over-dispersion patterning of plant individuals/ramets to relieve the tension of competition (see also figure 1 for this working hypothesis). To this end, we analysed local spatial pattern of ramets using the pair-correlation function (also known as the *g* function in spatial point pattern analysis [51]).

For the evaluation of ecosystem functioning, we are particularly interested in primary productivity and biodiversity, because they are among the most important aspects, and have been linked to spatial self-organization in regularly patterned systems [52,53]. For assessing primary productivity, we measured the change of dry total biomass (including both above- and below-ground biomass) of *S. mariqueter* during the growing season of 2017. Considering that benthic macroinvertebrates are a major component of biodiversity and play an essential role in the functioning in coastal salt marsh ecosystems [54], we collected data of the macrobenthos community with four replicates in the exposed and unexposed areas around the transplant experiments, respectively. Square plots sized 0.2 × 0.2 m^2^ were established at the centre and the edge of the vegetated areas containing high and low plant density, respectively. The plot size guaranteed the inclusion of a high number of species and individuals per species. We took all samples to the laboratory and collected all macrobenthos using 100 mesh sieves. All samples were immediately fixed in 5% formaldehyde solution and then stored in a refrigerator at a temperature of −20°C. The samples were then counted and identified at the species level. Then, we calculated the richness, abundance, evenness, and indices of β-diversity. For β-diversity, we conducted a pairwise comparison of all plots and extracted all values of replicated plots [55]. Note that the areas of the transplant experiments and field investigations (covering *ca* 0.1 km^2^ in total) were fully embedded in the area of the remote sensing analysis.

### (e) Statistical analyses

We used a generalized linear mixed model with a Gaussian distribution and Satterthwaite approximation of the degrees of freedom to analyse the correlation between the response variables and explanatory variable. In order to test the presence of SDF, we set location as the explanatory variable, biomass change and density change as the response variables. When analysing ecosystem functioning, we chose exposure and plant density as the explanatory variables, and chose biomass per individuals, shoot-to-root ratio, abundance, richness, and indices of β-diversity of macrobenthos as response variables. We used one-way ANOVA to test the effects of treatments on the response variables. We further used Tukey's honest significant difference post hoc analysis of variance for comparing the difference between the means of the levels of explanatory variables. As for indices of β-diversity, we used the Wilcoxon test with adjusted *p*-values to perform multiple median comparisons [56]. All statistical tests were implemented in R v. 3.4.2 [57], all the raw data and R script are available in the Dryad Digital Repository: https://doi.org/10.5061/dryad.b78n9r1 [58].

## 3. Results and discussion

### (a) Regular versus irregular patterning

**Figure 2.**
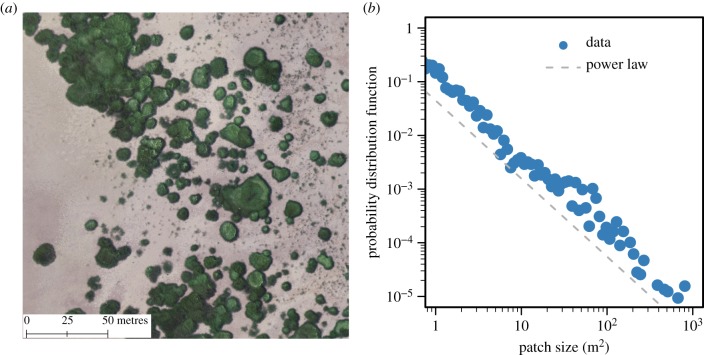
(*a*) A typically spatial distribution of patches showed an apparently irregular vegetation pattern in the studied intertidal salt marsh ecosystems. The vegetation patches (dark green) are extracted from the aerial photo taken by an unmanned aerial vehicle in July 2018. More data analysis is shown in electronic supplementary materials. (*b*) The power-law-like patch-size distribution (*b* = −1.45, *R*^2^ = 0.92, *p* < 0.001) confirmed the irregular pattern, as opposed by Gaussian distributions indicating regular patterns.

In the intertidal salt marshes that were studied, we observed apparently irregular vegetation patterns in *S. mariqueter* (figure 2 and electronic supplementary material, figure S1 for details), with patch size ranging from 1 to more than 100 m^2^. The irregularity of the patterning was rigorously confirmed by our analysis of patch-size distribution. As regular patterns would commonly have patches with similar sizes, one would expect a uniform- or Gaussian-like distribution of patches. Instead, our result clearly demonstrates that a scale-free distribution (following a power law, as characterized by frequent small patches and few large patches) is more plausible (figure 2;*b* = −1.45, *R*^2^ = 0.92, *p* < 0.001), suggesting that there is no ‘typical’ patch size (as expected to exist in a regular pattern) in the system. This makes a simple but effective way to reject regularity of spatial patterning. Patch-size distribution has been suggested as an important spatial signature for inferring system dynamics. For instance, a power-law-like distribution may suggest that the dynamics of the system are dominated by random perturbations [27,28]; a truncated power-law distribution may arise from a local-scale positive feedback (e.g. plant–plant facilitation) interacting with a global-scale stress factor (e.g. grazing) [29]. Unimodal distributions (dominated by single, typical patch sizes) have been linked with SDF [45]. However, recent studies have suggested that unimodal patterns can also arise from other processes, such as biological movement behaviours [16]. By showing that SDF does not necessarily give rise to unimodal patterns, our result further undermines the correspondence between SDF and unimodal distribution, and highlights that SDF can play a more complex role in pattern formation than previously thought.

### (b) Scale-dependent feedbacks

**Figure 3.**
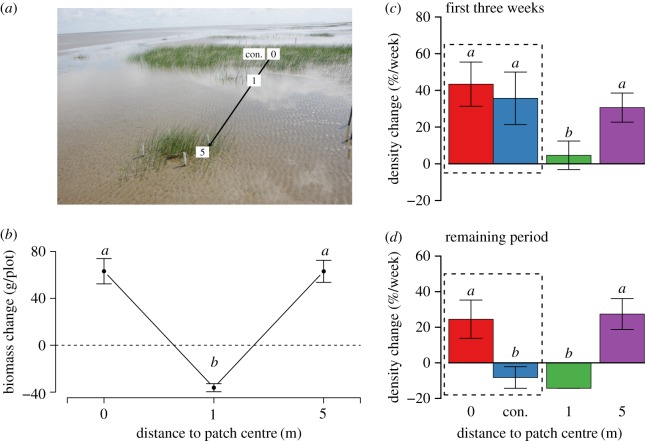
Results from the transplant experiments testing the scale-dependent feedbacks on *S. mariqueter* growth in terms of the biomass and density change versus spatial distances. (*a*) Short-range positive feedback is found within vegetation patches, as indicated by the net increase of total dry biomass (above- and below-ground biomass) per plot (mean ± s.e.), whereas long-range negative feedback is found in areas that neighbour the vegetation patches, as indicated by the net loss of biomass. Different letters above the bars indicate significant differences (*p* < 0.05). (*c,d*) Plant density change also indicates scale-dependent feedbacks during different periods. ‘Con.’ represents control treatments that exclude the competition effect by removal from neighbourhood above-ground biomass of transplant patches.

Performance of *S. mariqueter* markedly varied as a function of distance to the vegetation patches (figure 3*b*: *F*_2,9_ = 45.43, *p* < 0.001; figure 3*c*: *F*_3,20_ = 3.16, *p* < 0.05; figure 3*d*: *F*_3,20_ = 6.65, *p* = 0.003). On bare sediment about 5 m away from the vegetation patches, transplanted individuals appeared unaffected by the existing patches (figure 3*b*; *t* = −0.01, d.f. = 9, *p* > 0.99 for the comparison between the 0 m and 5 m treatments). In the within-patch locations, the transplanted individuals had *ca* 300% higher biomass increase and *ca* 40% higher survival rate than close to the patches (*t* = −8.26, d.f. = 9, *p* = 0.04 and *t* = −2.95, d.f. = 20, *p* = 0.04 for the comparison between the 0 m and 1 m treatments in figure 3*b* and *c*, respectively). The transplanted individuals in the control treatment (excluding intraspecific competition for light) did not show significant difference compared with those in the 0 m treatment in terms of density rate of change in the first three weeks (figure 3*c*; *t* = −0.53, d.f. = 20, *p* = 0.95), suggesting that plant-plant facilitation was sufficiently strong to cancel out the negative effect of light competition. Moreover, without the protection from neighbouring plants, the control treatment became more susceptible to wave impacts, showing declined densities during the remaining period after three weeks (figure 3*d*). For the 1 m treatment (at a short distance outside the patches), we observed the lowest survival and growth, as most transplanted individuals were scoured away after a whole growing season (figure 3*c,d*).

These contrasting plant performances can be explained by scale-dependent feedbacks. Physical stress caused by wave impact is usually the limiting factor for colonization and growth of plants in tidal front areas [59]. It has been repeatedly observed that the protection from neighbouring established plants can effectively ameliorate such an impact [31], making local plant-plant facilitation the prime mechanism underlying the enhanced performance of *S. mariqueter* within the vegetation patches. In contrast to this positive effect at the small scale, the formation of turbulence around vegetation patches can reinforce wave impact, largely preventing plant colonization at a larger spatial scale (such scale depends on the neighbouring vegetation patches). This long-range negative effect has been confirmed by previous studies based on models and field observations on hydrodynamic conditions next to vegetation patches in intertidal salt marshes [31,59]. Taken together, the short-range facilitation and long-range inhibition are coupled and self-reinforced by the colonization and growth of plants, thus exhibiting a clear SDF. This particular mechanism has only been reported for the formation of patchy vegetation dominated by the bunchgrass species *Spartina* spp. in a European coastal salt marsh [31]. While it makes intuitive sense that the *Spartina* spp. with rather dense ramets and high biomass can generate significant feedbacks, our results reveal that the much sparser and lower-statured *S. mariqueter* can also create a pronounced SDF (see also electronic supplementary material, figure S1 for photos of the vegetation and typical gullies that formed around the vegetation patches, as a clear sign of a strong hydraulic scouring force suggested by van Wesenbeeck *et al*. 2008 [31]).

Our *in situ* transplantation experiments provide clear evidence that SDF is an essential element of self-organization, and plausibly plays an important role in the irregular vegetation patterning. However, it remains an open question why SDF can be a driving process behind both regular and irregular vegetation patterns. Two possible explanations may exist. One explanation is that the SDF (especially the localized negative feedback next to vegetation patches originated from reinforced wave impact) is not strong or persistent enough to kill all seedlings, so that some seedlings would still have sufficient chance to survive on the inter-patch sediments. The relevance of a stochastic element contributing to the negative feedback can be demonstrated by a simple individual-based model for vegetation patterning in harsh environments—if environmental stochasticity is high, regular patterning can be disturbed even when SDF is active; and regular patterning can only arise when the negative feedback is sufficiently strong to create persistent inter-patch spacing [60]. Another possibility is that the SDF is highly anisotropic, preventing the formation of persistent direction. In our study ecosystems, the strength of the negative feedback can vary greatly across different directions, as gullies that are sometimes formed next to patches can be observed to have many different directions. In any case, it could well be that both mechanisms play a role. Elegant field experiments are needed to test and disentangle these mechanisms.

### (c) The shaping role of self-organization

**Figure 4.**
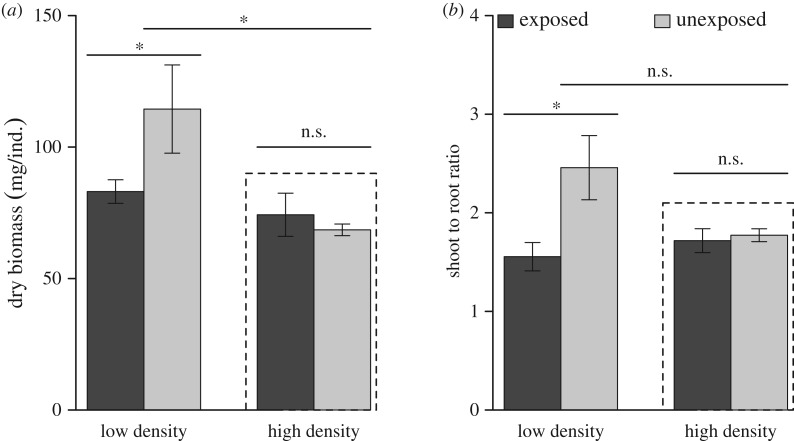
Primary productivity (*a*) and shoot-to-root ratio (*b*) of *S. mariqueter* at different levels of environmental stress (exposed versus unexposed) and plant density (high versus low) (mean ± s.e.). Asterisks above the bars indicate significant differences (*p* < 0.05), n.s., not significant. Strong effects of self-organization are found under high biomass density (dashed boxes).

How would ecosystems respond to the process of self-organization? Considering that local facilitation would inevitably increase local crowdedness, potentially intensifying resource competition between neighbouring plants, an intuitive speculation is that local spatial patterning of plant individuals (ramets) might shift towards over-dispersion in response to increasing density. However, our analysis based on the spatial pair-correlation function showed that the patterning did not significantly deviate from complete spatial randomness (electronic supplementary material, figure S2), suggesting the absence of micro-scale spatial structure. Instead, the field data support our second working hypothesis that plant functional traits are modified in the presence of self-organization. At low plant densities, the exposed areas yielded 33% lower biomass per individual plant than the unexposed areas, suggesting that wave impact can strongly reduce plant performance when self-organization is under-developed; in contrast, high-density plants (which reinforced self-organization) were immune to this stress (figure 4*a*; density: *F*_1,12_ = 8.02, *p* < 0.05). How can plant biomass be maintained even in the presence of synergetic negative effects from wave impact and local crowdedness? The answer may hinge on plant functional traits and strategies [40]. We observed *ca* 20% lower shoot-to-root ratios of high-density plants, indicating that more biomass was allocated underground in response to intensified local crowdedness and/or wave impact (figure 4*b*; exposure: *F*_1,12_ = 6.30, *p* < 0.05; exposure × density: *F*_1,12_ = 4.93, *p* < 0.05). This can be an efficient strategy, because it can alleviate competition for light between neighbouring individuals and better protect the plants against waves through a densified rhizome network. A reasonable speculation is that low-density plants exposed to wave impact tended to sprout more branches (electronic supplementary material, figure S4 and table S1) with a smaller angle (thus facilitating local clustering) against the stress, while high-density plants tend to branch less and expand between-node rhizome length to alleviate local competition.

**Figure 5.**
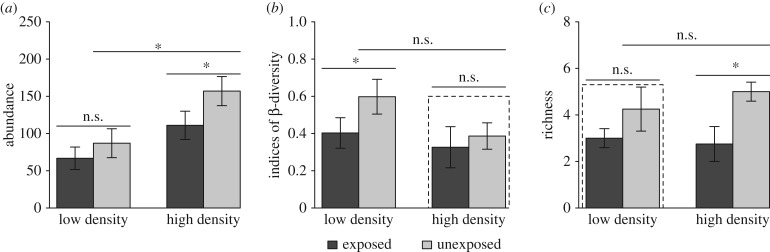
Abundance (*a*), β-diversity (*b*), and species richness (*c*) of macrobenthos at different levels of environmental stress (exposed versus unexposed) and plant density (high versus low) (mean ± s.e.). Asterisks above the bars indicate significant differences (*p* < 0.05), n.s., not significant. Strong effect of self-organization on β-diversity and richness are found under high- and low-density plants, respectively (dashed box).

As the ecosystem engineer in the intertidal front zone, *S. mariqueter* can facilitate the accumulation of sediments and change the physical conditions. Within the high-density vegetation patches, we found an almost twofold higher benthonic abundance (figure 5*a*; *F*_1,12_ = 9.68, *p* = 0.009), suggesting that well self-organized vegetation can indeed provide shelter against wave disturbance to benthic animals [61,62]. Such a sheltering effect can help to maintain benthonic abundance when stress is strong (as indicated by the insignificant difference between the exposed and unexposed areas). As unexposed areas have a *ca* 20% higher microbenthic species richness than exposed areas, the species richness of the macrobenthos is mostly dependent on intensity of wave stress rather than plant density (figure 5*c*; exposure: *F*_1,12_ = 8.2, *p* = 0.01). It suggests that harsh wave stress would inhibit the establishment of the macrobenthos. When looking at species turnover between the vegetation patches, we found exposed areas have about 26% lower β-diversity, probably because strong wave impact can largely filter out those species with lower sessility, thereby homogenizing species distribution at a larger scale. However, this environmental filtering effect can be cancelled out by the presence of self-organized vegetation, as evidenced by the similarity in β-diversity at high plant densities found at exposed and unexposed areas (figure 5*b*; Wilcoxon test, *Mdn* = 0.32 and 0.39, respectively, *p* = 0.69, *r* = −0.12 in high density; *Mdn* = 0.42 and 0.62, respectively, *p* = 0.03, and *r* = −0.62 in low density). Hence, the effect of spatial patterning on biodiversity is evident mostly at a larger spatial scale: the species richness of the unexposed area is indeed 28% higher than that of the exposed area, when putting together all investigated vegetation patches (figure 5*c*; exposure: *F*_1,12_ = 8.2, *p* = 0.01).

Note that there were no significant differences between exposed and unexposed treatments at high density in terms of biomass (figure 4*a*; *F*_1,6_ = 0.46, *p* = 0.53) and traits (figure 4*b*; *F*_1,6_ = 0.16, *p* = 0.70). Furthermore, biomass and traits in high density are similar to that in exposed low-density treatment (figure 4*a*, exposed: low density-unexposed: high density, *t* = −1.07, *p* = 0.72; exposed: low-density exposed: high density, *t* = −0.65, *p* = 0.92; figure 4*b*, exposed: low-density unexposed: high density, *t* = 0.81, *p* = 0.85; exposed: low-density exposed: high density, *t* = 0.60, *p* = 0.93). They suggest the existence of competition in this ecosystem. At high density, there is less wave impact but resource is shared among individuals and hence competition becomes the driver of biomass and traits. There is an obvious difference of wave disturbance between the exposed and unexposed area. Hence, the absence of differences of individual traits at high density directly support our hypothesis that self-organization can optimize the efficiency of nutrient utilization under strong competition and in stressful environments (figure 1, H4). Similarly, this self-organization functioning still hold on β-diversity and richness as shown in figure 5. The β-diversity is apparently maintained as the same level between the exposed high density and unexposed high-density treatments (figure 5*b*; Wilcoxon test, *Mdn* = 0.32 and 0.39, respectively; *p* = 0.69 and *r* = −0.12). Furthermore, the richness has no significant difference between the exposed low density and unexposed low-density treatments (figure 5*c*; *F*_1,6_ = 1.47, *p* = 0.27). A self-organized vegetation pattern can eliminate environmental filtering effects to improve the turnover rate of the macrobenthos, and alleviate wave stress to eliminate the difference of the richness of the macrobenthos in the sparse plant sheltered areas.

In support of our predictions, *S. mariqueter* exhibited specific dispersal strategies that are likely adaptive under local facilitation, especially when facing strong wave impact (see electronic supplementary material, figure S4 and table S1). In response to environmental stress, behavioural self-organization (organism movement can be involved or not) has been identified in many organisms, including foraging behaviour, pest/predator avoidance behaviour, and competition alleviation behaviour [20,39,48,63]. Recent evidence supports that behaviour and intelligence underpins fitness for all organisms, including plants [38,39,48]. Plants explore and exploit above- and below-ground environments by growth rather than movement, leading to a highly plastic root-to-shoot ratio and other traits in response to environmental changes. Spatial self-organization may be able to maintain the traits towards optimal utilization of nutrients when the environment deteriorates (e.g. intraspecific competition increases in our case, figure 4 and electronic supplementary material, figure S3). Interest in linking plant functional traits to behavioural self-organization is emerging [45,63].

Our results also raise the interesting question of whether enhanced ecosystem functioning is a direct effect of spatial self-organization *per se* or an indirect effect brought about by increased biodiversity resulting from self-organization (or both). Indeed, it has been predicted by the biodiversity–ecosystem–functioning (BEF) theory and has been repeatedly observed that increased biodiversity can stimulate the primary productivity of terrestrial, aquatic, and marine ecosystems [45,53,64]. Nevertheless, it seems unlikely that BEF theory can solely explain our results, because multispecies interactions (at the core of BEF theory) are largely lacking in the studied intertidal salt marsh ecosystem containing only a limited number of species. While our case study suggests that it makes more intuitive sense to attribute enhanced productivity to self-organization (of plants) that can create better microhabitats (for macrobenthos), further theoretical and empirical work are required to elucidate the relationship between biodiversity and self-organization at different scales [65].

In summary, our results reinforce the previous suggestion that SDF can give rise to irregular spatial patterns in coastal salt marsh vegetation [31], and that self-organization can enhance ecosystem functioning [3,61,66]. Our work extends the current line of research on self-organization from two viewpoints. We highlight that SDF can indeed play an important role in shaping ecosystem structure and functioning, for both irregular as well as regular patterns, and even when within-patch biomass is low (as in our study sites). This highlights the important implication that self-organization could be at play in a much wider range of ecosystems than previously documented. Finally, our work also helps to link between the so-far largely unconnected fields of self-organization theory and trait-based, functional ecology. Despite its limitations (for instance, we are not able to precisely address a number of important processes including the formation, interaction, and extinction of vegetation patches where water-sediment dynamics are involved), our work may serve as a starting point for building a more complete picture. Only through rigorous field experimentation in combination with thoughtful modelling and spatial pattern analyses will we be able to fully elucidate how those fundamental ecosystem aspects at different levels are inter-connected. We encourage future studies to expand this link towards a complete picture of understanding patterns, mechanisms, and consequences of self-organization across a comprehensive range of ecological scales.

## Supplementary Material

FigS1-S5

## Data accessibility

Data available from the Dryad Digital Repository at: https://doi.org/10.5061/dryad.b78n9r1 [58].

## References

[1] Golding I, Kozlovsky Y, Cohen I, Ben-Jacob E 1998 Studies of bacterial branching growth using reaction–diffusion models for colonial development. Physica A 260, 510–554. (10.1016/S0378-4371(98)00345-8)

[2] Be'er A, Zhang HP, Florin E-L, Payne SM, Ben-Jacob E, Swinney HL 2009 Deadly competition between sibling bacterial colonies. Proc. Natl Acad. Sci. USA 106, 428–433. (10.1073/pnas.0811816106) 19129489 PMC2626719

[3] Pringle RM, Doak DF, Brody AK, Jocque R, Palmer TM 2010 Spatial pattern enhances ecosystem functioning in an African Savanna. PLoS Biol. 8, e1000377 (10.1371/journal.pbio.1000377) 20520846 PMC2876046

[4] Bonachela JA, Pringle RM, Sheffer E, Coverdale TC, Guyton JA, Caylor KK, Levin SA, Tarnita CE 2015 Termite mounds can increase the robustness of dryland ecosystems to climatic change. Science 347, 651–655. (10.1126/science.1261487) 25657247

[5] Getzin S et al. 2016 Discovery of fairy circles in Australia supports self-organization theory. Proc. Natl Acad. Sci. USA 113, 3551–3557. (10.1073/pnas.1522130113) 26976567 PMC4822591

[6] Tarnita CE, Bonachela JA, Sheffer E, Guyton JA, Coverdale TC, Long RA, Pringle RM 2017 A theoretical foundation for multi-scale regular vegetation patterns. Nature 541, 398–401. (10.1038/nature20801) 28102267

[7] Klausmeier CA 1999 Regular and irregular patterns in semiarid vegetation. Science 284, 1826–1828. (10.1126/science.284.5421.1826) 10364553

[8] Rietkerk M, Dekker SC, de Ruiter PC, van de Koppel J 2004 Self-organized patchiness and catastrophic shifts in ecosystems. Science 305, 1926–1929. (10.1126/science.1101867) 15448261

[9] Juergens N 2013 The biological underpinnings of Namib desert fairy circles. Science 339, 1618–1621. (10.1126/science.1222999) 23539605

[10] Rietkerk M, Dekker SC, Wassen MJ, Verkroost AW, Bierkens MF 2004 A putative mechanism for bog patterning. Am. Nat. 163, 699–708. (10.1086/383065) 15122488

[11] van de Koppel J, Rietkerk M, Dankers N, Herman PMJ 2005 Scale-dependent feedback and regular spatial patterns in young mussel beds. Am. Nat. 165, E66–E77. (10.1086/428362) 15729660

[12] van de Koppel J, Gascoigne JC, Theraulaz G, Rietkerk M, Mooij WM, Herman PMJ 2008 Experimental evidence for spatial self-organization and its emergent effects in mussel bed ecosystems. Science 322, 739–742. (10.1126/science.1163952) 18974353

[13] Liu QX, Herman PMJ, Mooij WM, Huisman J, Scheffer M, Olff H, van de Koppel JV 2014 Pattern formation at multiple spatial scales drives the resilience of mussel bed ecosystems. Nat. Commun. 5, 5234 (10.1038/ncomms6234) 25335554

[14] Rietkerk M, Koppel JVD 2008 Regular pattern formation in real ecosystems. Trends Ecol. Evol. 23, 169–175. (10.1016/j.tree.2007.10.013) 18255188

[15] Meinhardt H 2009 The algorithmic beauty of sea shells. London, UK: Springer.

[16] Liu QX, Doelman A, Rottschafer V, de Jager M, Herman PMJ, Rietkerk M, van de Koppel J 2013 Phase separation explains a new class of self-organized spatial patterns in ecological systems. Proc. Natl Acad. Sci. USA 110, 11 905–11 910. (10.1073/pnas.1222339110) PMC3718087 23818579

[17] Liu Q-X, Rietkerk M, Herman PMJ, Piersma T, Fryxell JM, van de Koppel J 2016 Phase separation driven by density-dependent movement: a novel mechanism for ecological patterns. Phys. Life Rev. 19, 107–121. (10.1016/j.plrev.2016.07.009) 27478087

[18] Nicolis G, Prigogine I 1977 Self-organization in nonequilibrium systems: from dissipative structures to order through fluctuations. New York, NY: Wiley.

[19] Kauffman SA 1996 At home in the universe: the search for laws of self-organization and complexity. London, UK: Penguin.

[20] Camazine S, Deneubourg J-L, Franks NR, Sneyd J, Theraulaz G, Bonabeau E 2001 Self-organization in biological systems. Princeton, NJ: Princeton University Press.

[21] Sheth R, Marcon L, Bastida MF, Junco M, Quintana L, Dahn R, Kmita M, Sharpe J, Ros MA 2012 Genes regulate digit patterning by controlling the wavelength of a Turing-type mechanism. Science 338, 1476–1480. (10.1126/science.1226804) 23239739 PMC4486416

[22] Hiscock TW, Megason SG 2015 Orientation of Turing-like patterns by morphogen gradients and tissue anisotropies. Cell Syst. 1, 408–416. (10.1016/j.cels.2015.12.001) 26771020 PMC4707970

[23] Karig D, Martini KM, Lu T, DeLateur NA, Goldenfeld N, Weiss R 2018 Stochastic Turing patterns in a synthetic bacterial population. Proc. Natl Acad. Sci. USA 115, 6572–6577. (10.1073/pnas.1720770115) 29891706 PMC6042114

[24] Koppel JVD, Rietkerk M 2004 Spatial interactions and resilience in arid ecosystems. Am. Nat. 163, 113–121. (10.1086/380571) 14767841

[25] D'Odorico P, Laio F, Porporato A, Ridolfi L, Barbier N 2015 Noise-induced vegetation patterns in fire-prone savannas. J. Geophys. Res. Biogeosci. 112, G02021.

[26] Borgogno F, D'Odorico P, Laio F, Ridolfi L 2009 Mathematical models of vegetation pattern formation in ecohydrology. Rev. Geophys. 47, RG1005 (10.1029/2007RG000256)

[27] Pascual M, Roy M, Guichard F, Flierl G 2002 Cluster size distributions: signatures of self-organization in spatial ecologies. Phil. Trans. R. Soc. Lond. B 357, 657–666. (10.1098/rstb.2001.0983) 12079527 PMC1692977

[28] Guichard F, Halpin PM, Allison GW, Lubchenco J, Menge BA 2003 Mussel disturbance dynamics: signatures of oceanographic forcing from local interactions. Am. Nat. 161, 889–904. (10.1086/375300) 12858274

[29] Kéfi S, Rietkerk M, Alados CL, Pueyo Y, Papanastasis VP, ElAich A, de Ruiter PC 2007 Spatial vegetation patterns and imminent desertification in Mediterranean arid ecosystems. Nature 449, 213–215. (10.1038/nature06111) 17851524

[30] Xu C et al. 2015 Can we infer plant facilitation from remote sensing? A test across global drylands. Ecol. Appl. 25, 1456–1462. (10.1890/14-2358.1) 26552256 PMC4910861

[31] van Wesenbeeck BK, van de Koppel J, Herman PMJ, Bouma TJ 2008 Does scale-dependent feedback explain spatial complexity in salt-marsh ecosystems? Oikos 117, 152–159. (10.1111/j.2007.0030-1299.16245.x)

[32] Weerman EJ, Van Belzen J, Rietkerk M, Temmerman S, Kefi S, Herman PMJ, Van de Koppel J 2012 Changes in diatom patch-size distribution and degradation in a spatially self-organized intertidal mudflat ecosystem. Ecology 93, 608–618. (10.1890/11-0625.1) 22624215

[33] Ludwig JA, Coughenour MB, Liedloff AC, Dyer R 2001 Modelling the resilience of Australian savanna systems to grazing impacts. Environ. Int. 27, 167–172. (10.1016/S0160-4120(01)00078-2) 11697665

[34] Holling CS 1973 Resilience and stability of ecological systems. Annu. Rev. Ecol. Syst. 4, 1–23. (10.1146/annurev.es.04.110173.000245)

[35] Czárán TL, Hoekstra RF, Pagie L 2002 Chemical warfare between microbes promotes biodiversity. Proc. Natl Acad. Sci. USA 99, 786–790. (10.1073/pnas.012399899) 11792831 PMC117383

[36] Kerr B, Riley MA, Feldman MW, Bohannan BJM 2002 Local dispersal promotes biodiversity in a real-life game of rock-paper-scissors. Nature 418, 171–174. (10.1038/nature00823) 12110887

[37] Loreau M, Mouquet N, Gonzalez A 2003 Biodiversity as spatial insurance in heterogeneous landscapes. Proc. Natl Acad. Sci. USA 100, 12 765–12 770. (10.1073/pnas.2235465100) PMC240692 14569008

[38] de Paoli H, van der Heide T, van den Berg A, Silliman BR, Herman PMJ, van de Koppel J 2017 Behavioral self-organization underlies the resilience of a coastal ecosystem. Proc. Natl Acad. Sci. USA 114, 8035–8040. (10.1073/pnas.1619203114) 28696313 PMC5544259

[39] Pereira ML, Sadras VO, Batista W, Casal JJ, Hall AJ 2017 Light-mediated self-organization of sunflower stands increases oil yield in the field. Proc. Natl Acad. Sci. USA 114, 7975–7980. (10.1073/pnas.1618990114) 28696316 PMC5544258

[40] Violle C, Navas M-L, Vile D, Kazakou E, Fortunel C, Hummel I, Garnier E 2007 Let the concept of trait be functional! Oikos 116, 882–892. (10.1111/j.0030-1299.2007.15559.x)

[41] Young IM, Crawford JW 2004 Interactions and self-organization in the soil-microbe complex. Science 334, 1634–1637. (10.1126/science.1097394) 15192219

[42] von Hardenberg J, Kletter AY, Yizhaq H, Nathan J, Meron E 2010 Periodic versus scale-free patterns in dryland vegetation. Proc. R. Soc. B 277, 1771–1776. (10.1098/rspb.2009.2208) PMC2871858 20133355

[43] Casey ST, Cohen MJ, Acharya S, Kaplan DA, Jawitz JW 2015 On the spatial organization of the ridge slough patterned landscape. Hydrol. Earth Syst. Sci. Discuss. 12, 2975–3010. (10.5194/hessd-12-2975-2015)

[44] White EP, Enquist BJ, Green JL 2008 On estimating the exponent of power-law frequency distributions. Ecology 89, 905–912. (10.1890/07-1288.1) 18481513

[45] Johan VDK, Crain CM 2006 Scale-dependent inhibition drives regular tussock spacing in a freshwater marsh. Am. Nat. 168, E136–E147. (10.1086/508671) 17080356

[46] Vince SW, Snow AA 1984 Plant zonation in an Alaskan salt marsh: I. distribution, abundance and environmental factors. J. Ecol. 72, 651–667. (10.2307/2260074)

[47] Schwarz C, Ysebaert T, Zhu Z, Zhang L, Bouma TJ, Herman PMJ 2011 Abiotic factors governing the establishment and expansion of two salt marsh plants in the Yangtze estuary, China. Wetlands 31, 1011–1021. (10.1007/s13157-011-0212-5)

[48] Gruntman M, Groß D, Májeková M, Tielbörger K 2017 Decision-making in plants under competition. Nat. Commun. 8, 2235 (10.1038/s41467-017-02147-2) 29269832 PMC5740169

[49] Tjisse VDH, Bouma TJ, Nes EH, Van Johan VDK, Marten S, Roelofs JGM, Katwijk MM, Van Smolders AJP 2010 Spatial self-organized patterning in seagrasses along a depth gradient of an intertidal ecosystem. Ecology 91, 362–369. (10.1890/08-1567.1) 20392001

[50] Tjisse VDH, Eklof JS, Nes EH, Van Zee EM, Van Der Serena D, Weerman EJ, Han O, Britas Klemens E 2012 Ecosystem engineering by seagrasses interacts with grazing to shape an intertidal landscape. PLoS ONE 7, e42060 (10.1371/journal.pone.0042060) 22905115 PMC3414520

[51] Wiegand T, Moloney KA 2013 Handbook of spatial point pattern analysis in ecology. Boca Raton, FL: CRC Press.

[52] Liu QX, Weerman EJ, Herman PMJ, Olff H, van de Koppel J 2012 Alternative mechanisms alter the emergent properties of self-organization in mussel beds. Proc. R. Soc. B 279, 2744–2753. (10.1098/rspb.2012.0157) PMC3367779 22418256

[53] Tilman D, Isbell F, Cowles JM 2014 Biodiversity and ecosystem functioning. Ann. Rev. Ecol. Evol. Syst. 45, 471–493. (10.1146/annurev-ecolsys-120213-091917)

[54] Levin LA et al. 2001 The function of marine critical transition zones and the importance of sediment biodiversity. Ecosystems 4, 430–451. (10.1007/s10021-001-0021-4)

[55] Baselga A 2010 Partitioning the turnover and nestedness components of beta diversity. Glob. Ecol. Biogeogr. 19, 134–143. (10.1111/j.1466-8238.2009.00490.x)

[56] Benjamini Y, Hochberg Y 1995 Controlling the false discovery rate: a practical and powerful approach to multiple testing. J. R. Stat. Soc. B 57, 289–300. (10.1111/j.2517-6161.1995.tb02031.x)

[57] Team R.C. 2017 R: a language and environment for statistical computing. Vienna, Austria Available online at https://www.R-project.org/.

[58] Zhao L-X, Xu C, Ge Z-M, van de Koppel J, Liu Q-X. 2019 Data from: The shaping role of self-organization: linking vegetation patterning, plant traits and ecosystem functioning Dryad Digital Repository. (10.5061/dryad.b78n9r1) PMC6501680 30966990

[59] Van Oyen T, Carniello L, D'Alpaos A, Temmerman S, Troch P, Lanzoni S 2014 An approximate solution to the flow field on vegetated intertidal platforms: applicability and limitations. J. Geophys. Res. Earth Surf. 119, 1682–1703. (10.1002/2013JF003064)

[60] Xu C, Nes EHV, Holmgren M, Kéfi S, Scheffer M 2015 Local facilitation may cause tipping points on a landscape level preceded by early-warning indicators. Am. Nat. 186, E81–E90. (10.1086/682674) 26655579

[61] Angelini C, van der Heide T, Griffin JN, Morton JP, Derksen-Hooijberg M, Lamers LPM, Smolders AJP, Silliman BR 2015 Foundation species’ overlap enhances biodiversity and multifunctionality from the patch to landscape scale in southeastern United States salt marshes. Proc. R. Soc. B 282, 20150421 (10.1098/rspb.2015.0421) PMC4528541 26136442

[62] Angelini C, Griffin JN, van de Koppel J, Lamers LPM, Smolders AJP, Derksen-Hooijberg M, van der Heide T, Silliman BR 2016 A keystone mutualism underpins resilience of a coastal ecosystem to drought. Nat. Commun. 7, 12473 (10.1038/ncomms12473) 27534803 PMC4992128

[63] Trewavas A 2014 Plant behaviour and intelligence. Oxford, UK: Oxford University Press.

[64] Tilman D 1996 Biodiversity: population versus ecosystem stability. Ecology 77, 350–363. (10.2307/2265614)

[65] Hirt MR, Grimm V, Li Y, Rall BC, Rosenbaum B, Brose U 2018 Bridging scales: allometric random walks link movement and biodiversity research. Trends Ecol. Evol. 33, 701–712. (10.1016/j.tree.2018.07.003) 30072217

[66] Silliman BR, Schrack E, He Q, Cope R, Santoni A, van der Heide T, Jacobi R, Jacobi M, van de Koppel J 2015 Facilitation shifts paradigms and can amplify coastal restoration efforts. Proc. Natl Acad. Sci. USA 112, 14 295–14 300. (10.1073/pnas.1515297112) 26578775 PMC4655511

