# Supplementary material for: The shaping role of self-organization: linking vegetation patterning, plant traits and ecosystem functioning
Source: Proc Biol Sci. 2019 Apr 10;286(1900):20182859. doi: 10.1098/rspb.2018.2859 (PMC6501680; doi:10.1098/rspb.2018.2859)
Supplement: FigS1-S5 [file rspb20182859supp1.pdf]

The shaping role of self-organization: linking vegetation patterning, plant traits and ecosystem functioning

Li-Xia Zhao[1], Chi Xu[2,*], Zhen-Ming Ge[1], Johan van de Koppel[3], and Quan-Xing Liu[1,4,5*]

1. State Key Laboratory of Estuarine and Coastal Research, School of Ecological and Environmental Sciences, East China Normal University, Shanghai 200241, China
2. School of Life Sciences, Nanjing University, Nanjing 210023, China
3. Department of Estuarine and Delta Systems, Royal Netherlands Institute for Sea Research and Utrecht University, PO Box 140, 4400 AC Yerseke, The Netherland
4. Shanghai Key Lab for Urban Ecological Processes and Eco-Restoration & Tiantong National Station for Forest Ecosystem Research, School of Ecological and Environmental Sciences, East China Normal University, Shanghai 200241, China
5. Center for Global Change and Ecological Forecasting, School of Ecological and Environmental Science, East China Normal University, 200241 Shanghai, China;

DOI:10.1098/rspb.2018.2859

This Supplemental Materials include the following contexts:

1. **Description of transplanting experiments**
2. **Statistical analyses**
3. **Supplemental Figures S1 to S5**
4. **Supplemental Table S1**

1. Description of transplanting experiments

SDF has been commonly observed for shaping patchy vegetation structure in stressful environments such as coastal salt marshes [31,45]. In general, it is to fully solidity the existence of SDF in the field experiments, as patch development is a long-term process. Transplanting experiments can provide so-far the most convincing evidence *in situ* on the existence of SDF [31]. For example, by comparing the growth of transplanted plant units between inside (short-range interactions) and outside (long-range interactions) established vegetation patches, van Wesenbeeck et al. 2008 [31] demonstrated enhanced performance within patches while suppressed growth in the sediments next to the patches for the pioneer plant species *Spartina angilica* in a Dutch intertidal salt-marsh ecosystem, indicating short-range positive feedback and long-range negative feedback. In this study we conducted two-year (in 2017 and 2018) transplant experiments that are similar with that of van Wesenbeeck et al. 2008 [31], to test if SDF is present for shaping *S. mariqueter* patches in our study site. Specifically, In the 2017 experiment, we selected 6 pairs of neighboring vegetation patches in the front zone for the colonization of *S. mariqueter.* For each patch pair, we transplanted small *S. mariqueter* at the inter-patch gap and around the centers of both patches along a straight line (representing three treatments). For all treatments, we transplanted *S. mariqueter* (obtained from neighboring homogeneous *S. mariqueter* vegetated areas) within a consistent sediment sample volume of $50 \times 50 \times 20$ cm^3 in May, 2017 and June, 2018, respectively. All transplanting plots were flagged with PVC tubes at their corners. Subject to strong wave disturbance, only 4 out of 6 sets of transplanting plots had flags remained intact at the end of the growing season in the 2017 experiment and hence only 4 replicates remained for the statistical analysis (n = 4). In the 2018 experiment, we transplanted *S. mariqueter* at the center (0 m), close to (1 m) and faraway from patch (5 m), and added a

DOI:10.1098/rspb.2018.2859

control treatment to account for the potential influence of competition effect from neighboring plants. The control treatment is meant to account for intraspecific competition of *S. mariqueter*. In the studied intertidal salt marsh where nutrient supply is ample [46, 47], competition for light may play an important role in shaping vegetation structure [45]. In response to light competition, plants may promote vertical growth and shade tolerance to enhance their competitiveness, or alleviate the tension of competition through lateral growth [48]. To exclude the potential influence of light competition on the test of SDF, we implemented the control treatment next to the within-patch (0 m) treatment, close to the patch center. We removed the aboveground part of the neighboring plants within a distance of 20 cm to the borders of the transplanted plots per two weeks (see figure S3c). Every week we measured stems density within the transplanted plots at the three distances (i.e., 0 m, 1 m and 5 m) as well as within the control treatment plots. After the 2018 experiment, 4 out of 6 replicate plots remained intact ($n = 4$ for statistical analysis). In September, 2017 and August, 2018, all aboveground and belowground biomass within the plots were harvested before being scoured away or die out, and then dried to constant weights at 60 °C (dried in the oven for 7 days). The results from the 2018 and 2017 experiments are shown in figure 3 and S3 respectively.

2. Statistical analyses

We used a generalized linear mixed model with a Gaussian distribution and Satterthwaite approximation of the degrees of freedom to analyze the correlation between the response variables and explanatory variable. In order to test the presence of SDF, we set location as the explanatory variable, biomass change and density change as the response variables. When analyzing ecosystem functioning, we choose exposure and plant density as the explanatory variables, and choose biomass per individuals, shoot-to-root ratio, abundance, richness and indices of β-diversity of macro benthos as response variables. We conducted Shapiro-Wilk tests to check normality and Levene's test for homogeneity of the variances of the residuals. We used one-way ANOVA to test the effects of treatments on the response variables, including biomass change in the transplant experiments and transformed normalized density change during first three weeks (square root transformation $\sqrt{x}$), then used ANOVA to test the effects of exposure, plants density and their interplay on biomass change per individual, shoot-to-root-ratio and abundance and transformed richness ($\log(x)$) of macro benthos. We further used Tukey's honest significant difference post-

DOI:10.1098/rspb.2018.2859

hoc analysis of variance for comparing the difference between the means of the levels of explanatory variables. As for indices of β-diversity, we used Wilcoxon test with adjusted p-values to perform multiple median comparisons [56]. We used Levene's test to check the assumption of variances are equal for all samples in terms of density change with different temporal phases.

Variables were log- or square root-transformed as needed to meet normality assumptions. All statistical tests were implemented in R version 3.4.2 [57], all the raw data and R script are available in the Dryad Digital Repository (https://doi.org/10.5061/dryad.b78n9r1).

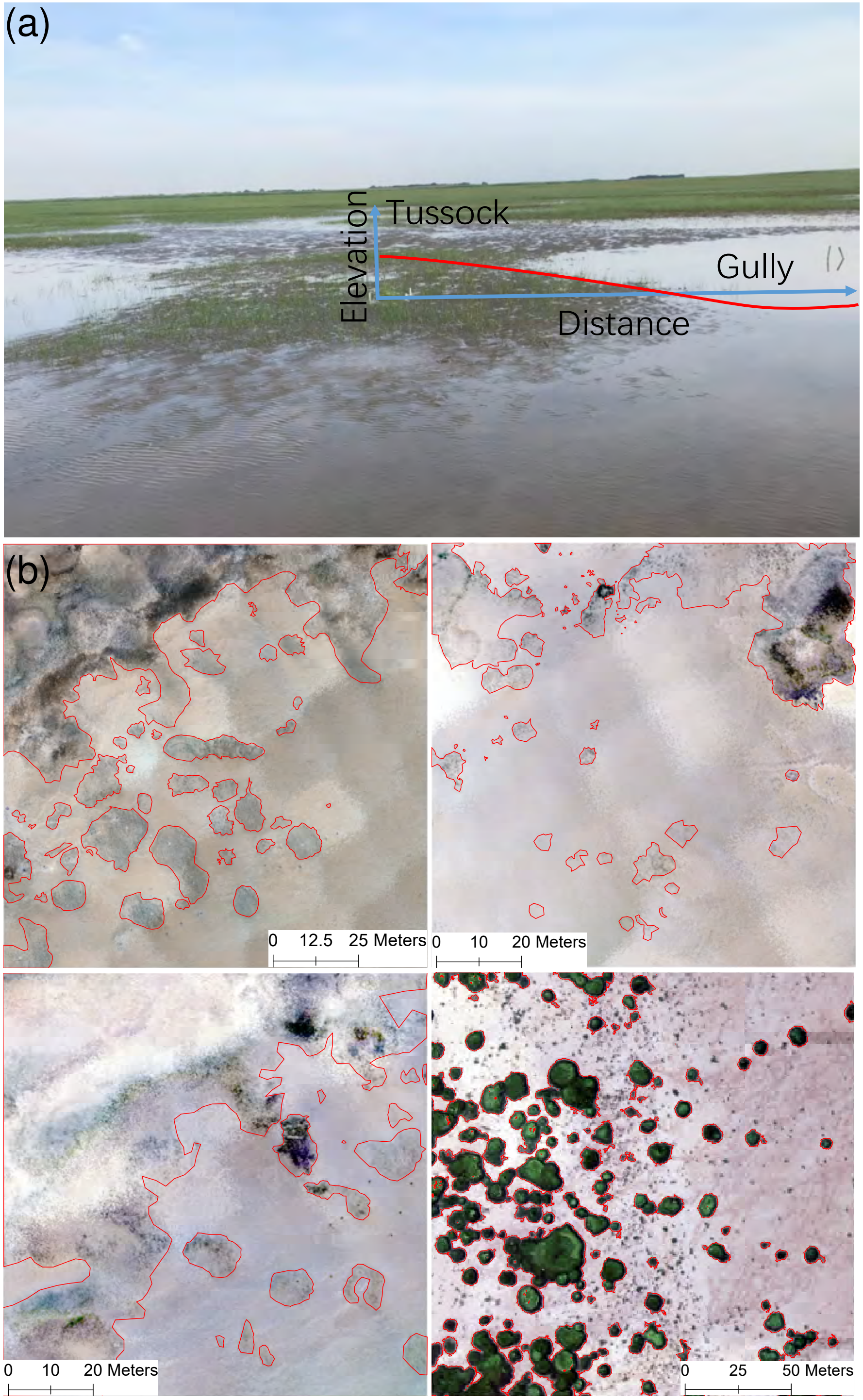

Figure S1. (a) Schematic representation of expected feedback and microtopography at different distances from patch center. (b) Four of all selected square samples of true color aerial image in Chongming Island. Vegetation patch was extracted by visual interpretation for calculating patch size distribution.

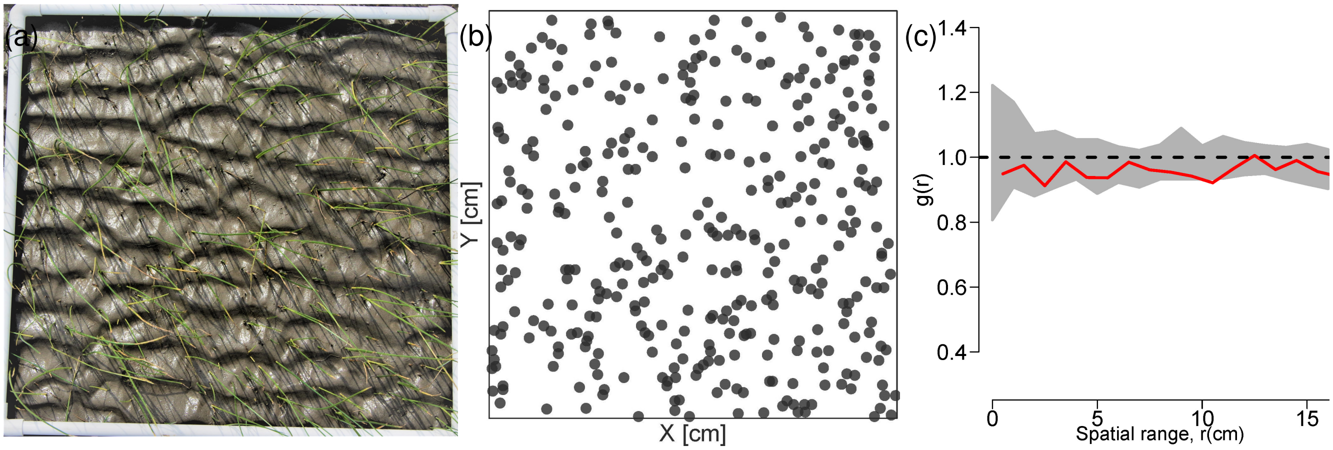

Figure S2. Point patterns of S. mariqueter. Observed (a) and extracted (b) point patterns of *S. mariqueter* derived from digital photographs which were used to calculate. (c) Results from application of the Ripley's K-test, *g(r)*, to an observed pattern of 371 individuals within a 50×50 cm^2 observation window for determining randomness of spatial point data. Gray regime represent confidence intervals (95%), solid red line represent *g(r)*-values and black dashed line represent expect values.

DOI:10.1098/rspb.2018.2859

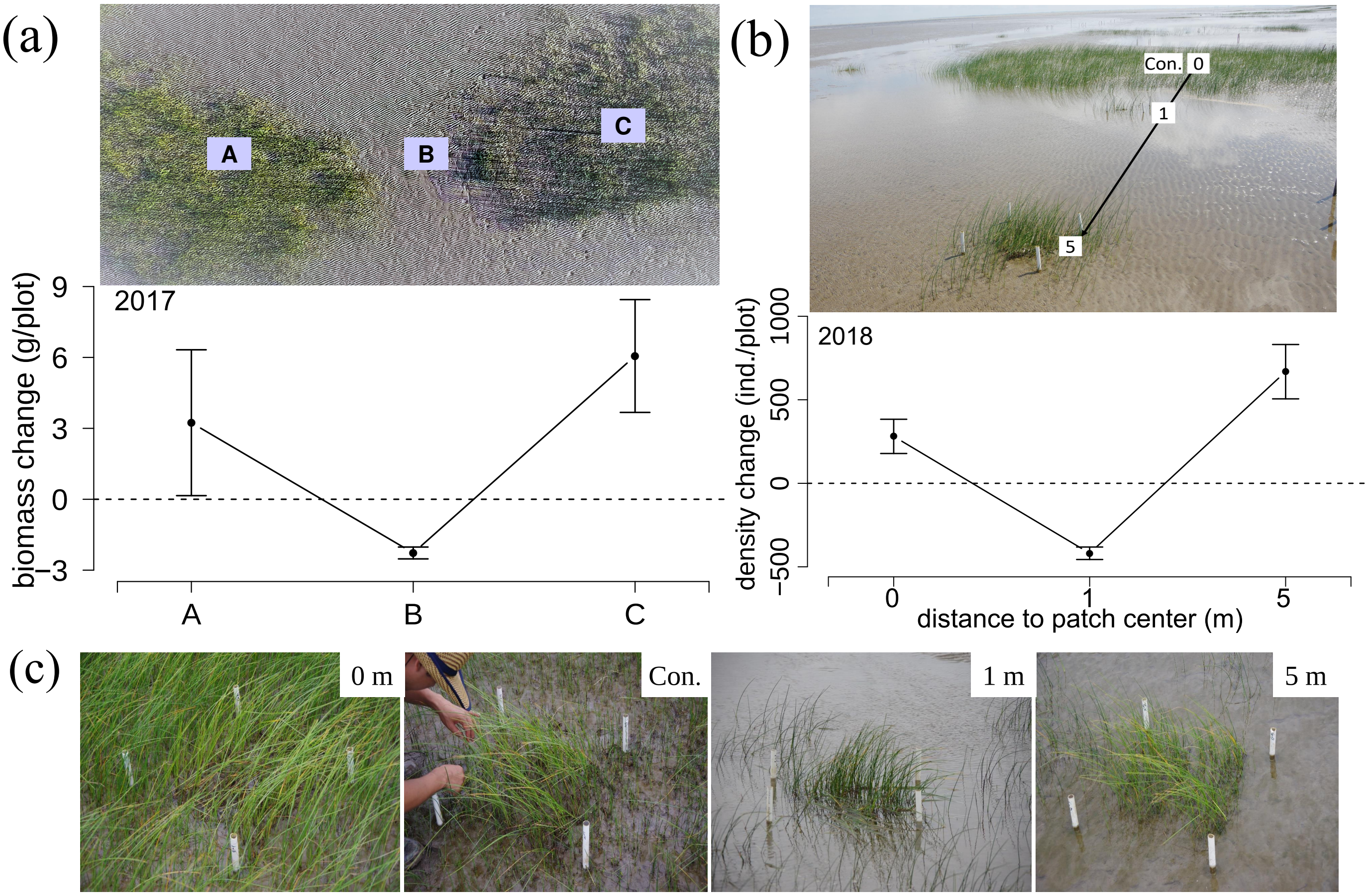

Figure S3. Results from the transplanting experiment made in 2017 (a) and in 2018 (b) demonstrate scale-dependent feedbacks on S. *Mariqueter*.

DOI:10.1098/rspb.2018.2859

(a)

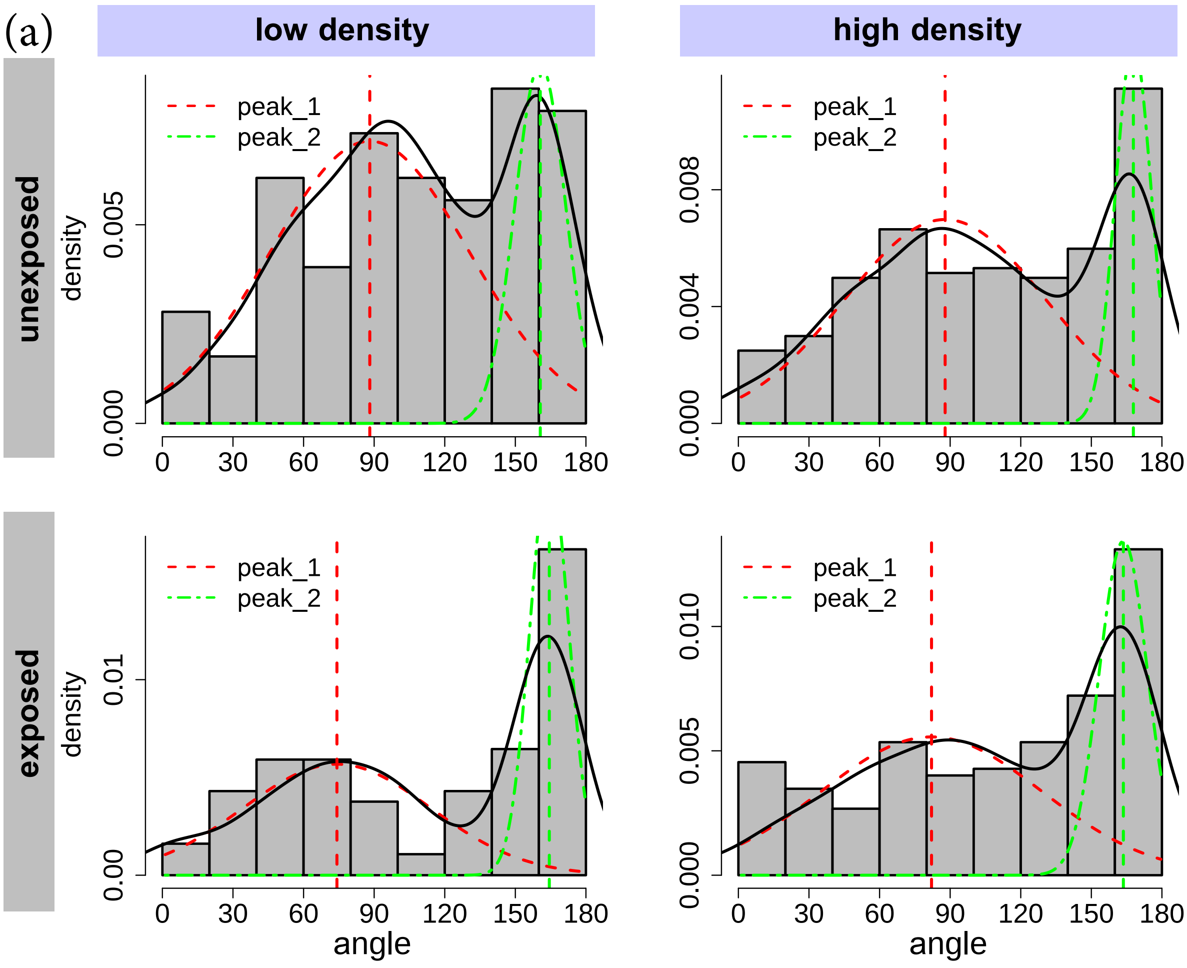
low density
high density
unexposed
exposed
density
peak_1
peak_2
0.000
0.005
0.004
0.008
0.00
0.01
0.010
0 30 60 90 120 150 180
angle

(b)

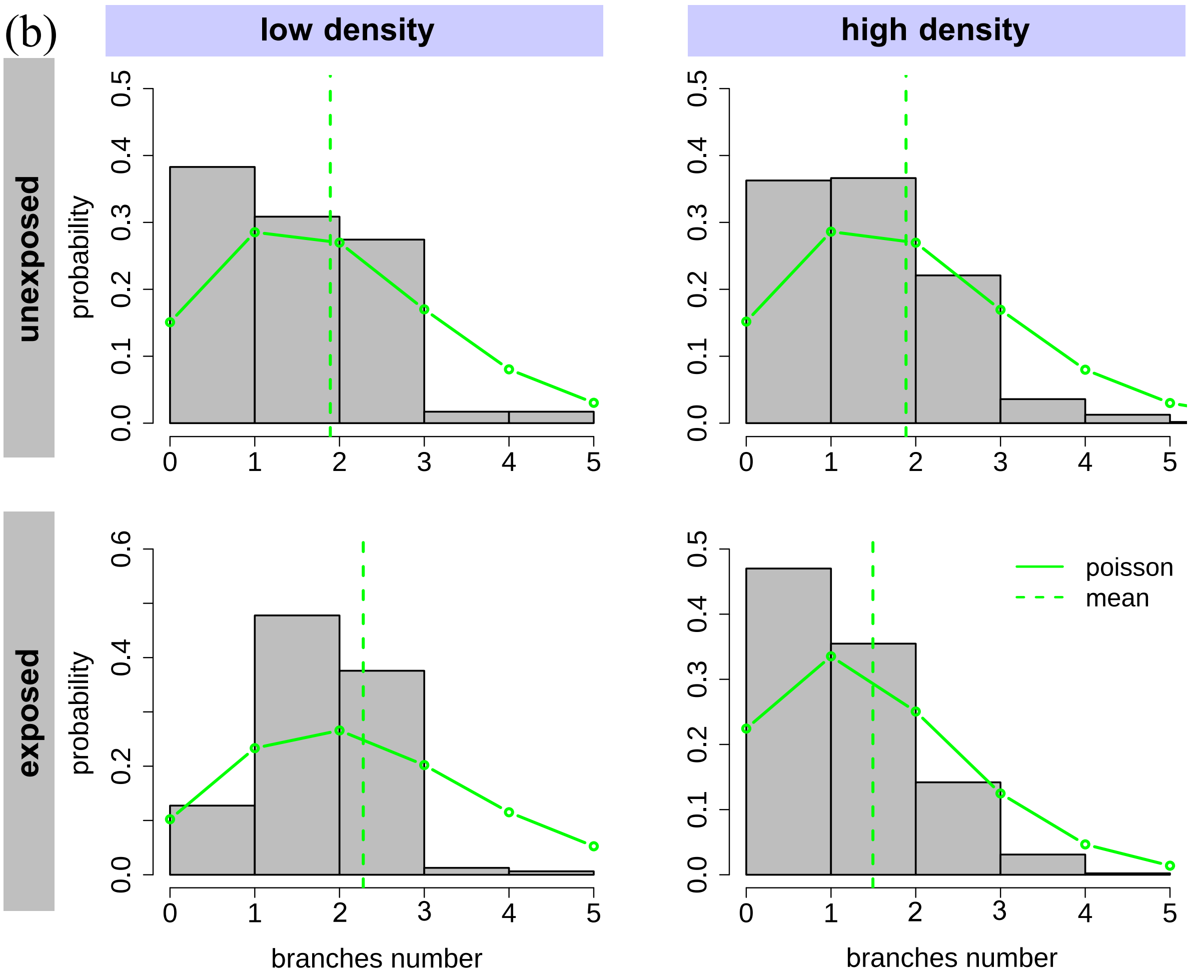
low density
high density
unexposed
exposed
probability
probability
poisson
mean
branches number
branches number

(c)

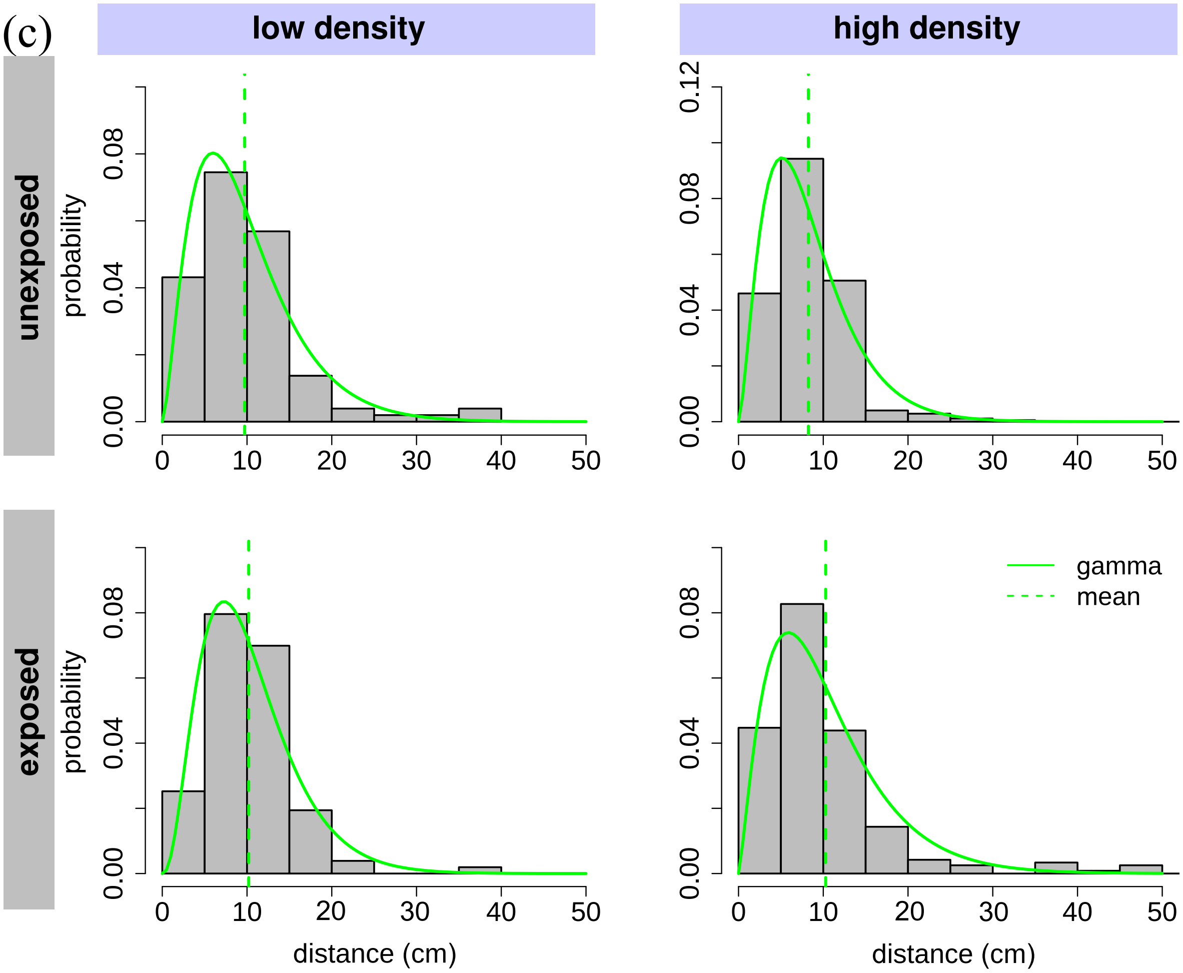

Figure S4. The probability density function of (a) angle, (b) branches number and (c) connection distances in different environmental stresses. Unexposed environment (upper panel) with low plants density (left) and high plants density (right); Exposed environment (lower panel) with low plants density (left) and high plants density (right).

DOI:10.1098/rspb.2018.2859

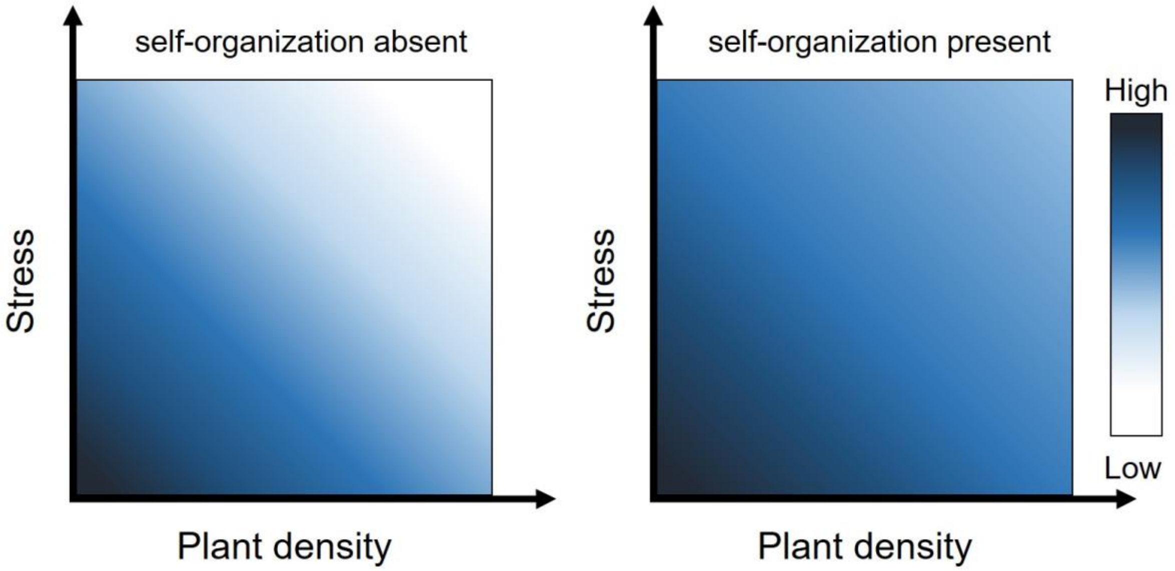

Figure S5: A conceptual illustration for the effect of vegetation self-organization on saltmarsh ecosystem functioning (the light-dark color gradient indicates low-high functioning). Increasing wave stress and plant density tend to reduce plant productivity and homogenize small-scale macro benthos species composition (lower β-diversity) (left), whereas these negative effects can be relieved in the presence of self-organization (right).

DOI:10.1098/rspb.2018.2859

Table S1: Significance of treatment effects on rhizome network parameters in terms of angle, distance and branches number. W represents the sum of the signed ranks, r indicates effect size.

Rhizome network	Factors	Parameters		
		W	p-value	r
angle	density	51891	**0.004**	-0.10
	exposure	86721	**0.001**	-0.11
distance	density	44578	0.88	-0.006
	exposure	58558	0.20	-0.049
branches number	density	134110	**<0.001**	-0.15
	exposure	205220	**0.01**	-0.07

DOI:10.1098/rspb.2018.2859
